# Analysis of two questionnaires on quality of life of Chronic Obstructive Pulmonary Disease patients*

**DOI:** 10.1590/1518-8345.2624.3148

**Published:** 2019-07-18

**Authors:** Ana Folch Ayora, Loreto Macia Soler, Agueda Cervera Gasch

**Affiliations:** 1 Universitat Jaume I, Facultad de Ciencias de la Salud, Castellón de la Plana, Comunidad Valenciana, Espanha; 2 Universidad de Alicante, Facultad de Ciencias de la Salud, Alicante, Comunidad Valenciana, Espanha

**Keywords:** Quality of Life, Pulmonary Disease Chronic Obstructive, Lung Diseases, Obstructive, Hospital Care, Nursing Assessment, Lung Disease, Qualidade de Vida, Doença Pulmonar Obstrutiva Crônica, Pneumopatias Obstructivas, Assistência Hospitalar, Avaliação em Enfermagem, Pneumopatias, Calidad de Vida, Enfermedad Pulmonar Obstructiva Crónica, Enfermedades Pulmonares Obstructivas, Atención Hospitalaria, Evaluación en Enfermería, Enfermedades Pulmonares

## Abstract

**Objective:**

to evaluate the efficacy of quality of life questionnaires *St. George Respiratory Questionnaire* and *Chronic Obstructive Pulmonary Disease Assessment Test* in patients with chronic obstructive pulmonary disease based on correlation and agreement analyses, and identify the most effective tool to assess their quality of life.

**Method:**

cross-sectional cohort study with patients hospitalized in a Spanish hospital for exacerbation of chronic obstructive pulmonary disease. Health-related quality of life was assessed with both questionnaires. The correlation and the agreement between the questionnaires were analyzed, as well as the internal consistency. Associations were established between the clinical variables and the results of the questionnaire.

**Results:**

one hundred and fifty-six patients participated in the study. The scales had a correlation and agreement between them and high internal consistency. A higher sensitivity of the *Chronic Obstructive Pulmonary Disease Assessment Test* was observed for the presence of cough and expectoration.

**Conclusion:**

the questionnaires have similar reliability and validity to measure the quality of life in patients with acute chronic obstructive pulmonary disease, and the *Chronic Obstructive Pulmonary Disease Assessment* Test is more sensitive to detect cough and expectoration and requires a shorter time to be completed.

## Abstract (pt)

**Objetivo:**

avaliar a eficácia entre os questionários de qualidade de vida *St. George Respiratory Questionnaire* e *Chronic Obstructive Pulmonary Disease Assessment Test* em pacientes com doença pulmonar obstrutiva crônica a partir da análise de correlação e concordância, bem como identificar a ferramenta mais eficaz para avaliar sua qualidade de vida.

**Método:**

estudo analítico de coorte transversal com pacientes internados em um hospital espanhol para exacerbação de doença pulmonar obstrutiva crônica. A qualidade de vida relacionada à saúde foi avaliada com os dois questionários. Analisaram-se a correlação e a concordância entre ambos, bem como a consistência interna. As associações foram estabelecidas entre as variáveis clínicas e os resultados do questionário.

**Resultados:**

participaram 156 pacientes. Ambas as escalas mostram correlação e concordância entre elas e alta consistência interna. Uma maior sensibilidade do *Chronic obstructive pulmonary disease Assessment Test* foi observada para detectar a presença de tosse e expectoração.

**Conclusão:**

ambos os questionários têm a mesma confiabilidade e validade para medir a qualidade de vida em pacientes com doença pulmonar obstrutiva crônica aguda, sendo que o *Chronic Obstructive Pulmonary Disease Assessment* é mais sensível para detectar a tosse e a expectoração e com um tempo de preenchimento mais curto.

## Abstract (es)

**Objetivo:**

evaluar la efectividad entre los cuestionarios de la calidad de vida St. George Respiratory Questionnaire y Chronic obstructive pulmonary disease Assessment Test en pacientes con enfermedad pulmonar obstructiva crónica a partir del análisis de su correlación y concordancia, e identificar la herramienta más efectiva para evaluar su calidad de vida.

**Método:**

estudio analítico transversal en pacientes ingresados en un hospital español por exacerbación de la enfermedad pulmonar obstructiva crónica. Se estudió la calidad de vida relacionada con la salud evaluada con los dos cuestionarios. Se analizó la correlación y concordancia entre ambos, así como su consistencia interna. Se establecieron asociaciones entre las variables clínicas y los resultados del cuestionario.

**Resultados:**

participaron 156 pacientes. Ambas escalas muestran correlación y concordancia entre ellas y consistencia interna elevada. Se observa una mayor sensibilidad del cuestionario Chronic obstructive pulmonary disease Assessment Test para detectar la presencia de tos y expectoración.

**Conclusión:**

ambos cuestionarios presentan la misma fiabilidad y validez para medir la calidad de vida en pacientes con enfermedad pulmonar obstructiva crónica agudizada, siendo el Chronic obstructive pulmonary disease Assessment Test más sensible para detectar tos y expectoración y con un tiempo de cumplimentación más breve.

## Introduction

Chronic obstructive pulmonary disease (COPD) is a common disease and 80 million people suffer from the severe or moderate form of this disease^(^ ^1^ ^–^ ^2^ ^)^. COPD is an avoidable and treatable disease^(^ ^1^ ^)^. Its diagnosis is established by the presence of persistent respiratory symptoms, such as dyspnea, cough and sputum production. The test to diagnose COPD is spirometry (forced expiratory volume ratio in the first second (FEV1)/forced vital capacity (FVC) less than 70 after bronchodilation)^(^ ^1^ ^)^.

Despite being an unknown disease, it is the third leading cause of death worldwide in developed countries after cardiac and oncological diseases^(^ ^1^ ^–^ ^3^ ^)^. Continuous reduction in lung function is a result of the progression of the disease. This situation worsens during periods of exacerbation, which happens once or twice a year. The treatment requires hospitalization to control the symptoms, in a large number of patients^(^ ^4^ ^)^, implying a high cost for the management of the pathology^(^ ^5^ ^)^. The exacerbations accelerate the continuous reduction of lung function, reducing the Health Related Quality of Life (HRQL) of patients.

HRQL is the personal evaluation made by an individual about the suffering caused by the effects of an illness or by the application of a treatment in various areas of life, especially the consequences on their physical, emotional or social well-being^(^ ^6^ ^)^.

HRQL in chronic respiratory patients is a good indicator of disease severity, being significantly related to the frequency of exacerbations^(^ ^7^ ^)^, and its serial assessment can act as an indicator of the onset of an exacerbation^(^ ^8^ ^)^. Moreover, it is a good independent predictor of mortality^(^ ^9^ ^)^.

Therefore, HRQL measurement of chronic respiratory patients is part of the routine evaluation of the results of therapeutic interventions^(^ ^8^ ^)^ performed by all health professionals, be them physicians, nurses, physiotherapists or psychologists, among others, in order to know the efficacy of the treatment adopted. Thus, HRQL assessment should be multidimensional in order to provide a better understanding and monitoring of the severity of the disease^(^ ^10^ ^–^ ^12^ ^)^ through valid and reliable scales^(^ ^13^ ^)^. Nursing is one of the groups that most use these two scales.

In order to measure HRQL, several questionnaires, either generic and specific, have showed to have optimal psychometric properties of reliability and validity to be used in COPD patients, with specific questionnaires being the most sensitive to changes during disease progression^(^ ^8^ ^)^.

Among the specific questionnaires, there are several that present reliability, validity, precision, consistency and sensitivity to changes, and are widely used to evaluate HRQL in patients with respiratory diseases. They have been adapted to different languages, but with different extensions, accessibility, ease of calculation of indices and filling time^(^ ^14^ ^)^. These characteristics may influence the gathering of information, especially if the patient needs to complete the questionnaire.

According to the recommendations of the Spanish Society of Respiratory Diseases (SEPAR) and international research^(^ ^15^ ^)^, the most used questionnaires are the *St. George Respiratory Questionnaire* (SGRQ) and the *Chronic Obstructive Pulmonary Disease Assessment Test* (CAT). The SGRQ^(^ ^16^ ^)^ validated in Spanish^(^ ^17^ ^–^ ^18^ ^)^ is the most used questionnaire in the population with respiratory diseases^(^ ^17^ ^,^ ^19^ ^)^, and it is also validated for administration via telephone call^(^ ^20^ ^)^. The SGRQ has 50 items distributed into three categories - symptoms, activity, and impact - with 76 weighted responses, and requires 10 minutes for completion^(^ ^17^ ^)^. Each item has an empirically derived weight, and a score has to be calculated.

The CAT questionnaire, recommended by the SEPAR, is aimed at evaluating HRQL in patients with a diagnosis of COPD. The instrument had initially 21 items^(^ ^21^ ^)^ which were later reduced to 8; a total score is obtained from the sum of these items^(^ ^22^ ^)^.

The SGRQ and the CAT present reliability, validity and sensitivity to changes during acute exacerbations^(^ ^23^ ^–^ ^25^ ^)^. The Cronbach's alpha of the SGRQ is 0.94 (symptoms: 0.72, activity: 0.89, impact: 0.89) and of the CAT is 0.88, and the intraclass correlation coefficient of the SGRQ and CAT is 0.9 and 0.8, respectively^(^ ^22^ ^)^.

A literature review revealed a significant correlation between the SGRQ and the CAT in a population with a diagnosis of COPD in primary care centers^(^ ^21^ ^–^ ^22^ ^,^ ^26^ ^–^ ^27^ ^)^. Likewise, it was observed that there was a correlation between the two scales in the hospital setting in patients with stable COPD^(^ ^23^ ^)^, where there is also a correlation between the two questionnaires, although CAT is of much faster and easier application.

No studies were found to investigate the best questionnaire to evaluate HRQL in the hospital setting in patients with exacerbated COPD, as confirmed in the Spanish guide to COPD patient care^(^ ^28^ ^)^. This is one of the most important moments to evaluate HRQOL, that is, to verify the effectiveness of the treatment provided, as well as to administer the help and resources necessary to train patients before discharge.

Despite the interest generated by the study of HRQOL, there is only consensus in the literature about the use of the CAT in non-acute stages^(^ ^29^ ^)^ and primary care centers^(^ ^22^ ^)^, while no consensus exist on the most appropriate choice to evaluate the HRQOL in hospitalized patients with exacerbated condition.

Therefore, the main objective of this study is to evaluate the efficacy of the SGRQ and CAT questionnaires to evaluate quality of life based on the analysis of their correlation and agreement, and to identify which is the simplest tool to evaluate the quality of life of hospitalized patients with severe exacerbation of COPD.

## Method

A cross-sectional study with patients admitted to the General University Hospital of Castellón (HGUCS) (Spain) was carried out between February 2014 and May 2016, in which the SGRQ and CAT were applied within the first five days of admission.

The study population was patients with exacerbation of COPD hospitalized in the HGUCS during the period of study. The sample size was 150 patients who had been diagnosed with severe COPD exacerbation during the 27-month study period, based on the annual mean of 229 admitted patients diagnosed with this condition, a 95% confidence, and a reposition rate of 22%, as based on the literature consulted^(^ ^30^ ^)^. Patients diagnosed with COPD exacerbation (ICD 491.2) were included on the basis of a history of smoking (active or previous) of at least 20 packs a year, along with the presence of obstruction of the airway flow defined as FEV1/FVC below 70 after bronchodilation, voluntarily decision to participate in the study after receiving explanations and after understanding the objective of the study. All patients who were unable to communicate due to physical or mental disabilities, terminal patients with life expectancy less than six months according to clinical criteria, and patients who met the criteria but rejected the invitation to share in the study were excluded.

The variable studied was the HRQOL measured by the SGRQ and CAT. Questionnaire *St. George Respiratory Questionnaire* (SGRQ), composed of 50 items divided into three dimensions: symptoms of respiratory pathology (eight questions); activities that are limited in daily life (16 questions); and impact, which refers to the social and psychological functioning that can change the patient's lifestyle (26 questions). The sum of the three dimensions results in a total score between zero and 100. Higher scores are indicative of poorer quality of life. A calculator is used for calculation^(^ ^31^ ^)^.

The CAT^(^ ^13^ ^)^ consists of eight questions related to cough, phlegm, chest tightness, breathlessness in activities of daily living, activity limitation at home, confidence leaving home, sleep, and energy. The score interval of each element varies between zero and five, with a maximum score of 40^(^ ^22^ ^)^. According to the total CAT scores and the revised literature, patients can be classified into the following categories: 1-10 low impact; 11-20 average impact; 21-30 high impact; 31-40 very high impact^(^ ^13^ ^,^ ^27^ ^)^.

The control variables were divided into sociodemographic variables: sex, age, and schooling; clinic variables: dyspnea, through the *Medical Research Council* (MRC)^(^ ^32^ ^)^, tcough, expectoration, wheezing, drowsiness, fever, need to sit, and edema (presented as dichotomous variables with yes/no answers), and pain through a visual analogue scale (VAS) with a score ranging from 0 to 10. The psychological variables (anxiety and depression) were studied using the HAD questionnaire (33-34) from the Hospital Anxiety and Depression Scale and, finally, the level of dependence was analyzed using the Barthel index^(^ ^35^ ^)^.

The data collection procedure was developed within the scope of a therapeutic education program called Aprendepoc. This is a randomized controlled trial with masked data analysis, with no blinding in the allocation of participants. With two task groups, the Intervention Group (IG) consisted of four group educational sessions, telephone follow-up, and delivery of informative leaflets; and the Control Group (CG) whose intervention was based on conventional care, taking into account the standard care provided to all patients without being included in the project (without group educational sessions, telephone follow-up, or delivery of additional documents).

The project was evaluated at admission (from the 3rd day of hospitalization) and at three months from the time of inclusion, although for this investigation only the data corresponding to the screening (beginning of the study) were used, because the sample was homogeneous at the time of recruitment. Our results are not affected by the performance of the Aprendepoc program.

To recruit patients, once a week, the main investigator conducted searched for all patients admitted to the hospital who met the inclusion criteria using a *software* tool called “integration”. This search allowed to locate the number of the room and the number of days of hospital stay. This was the basis to select only patients hospitalized for more than two days, because the patients had limitations to respond to the questionnaires at the day of admission. The questionnaires were applied and control variables were obtained through a structured interview, followed by self-completion of the HAD, Barthel, CAT and SGRQ. For the statistical study of sociodemographic variables, measures of central tendency were used. The results were presented as percentages, mean (x) and standard deviation (SD). To determine the correlation between the questionnaires, a factorial analysis of motifs allowed to establish eight CAT questions in three dimensions: CAT_symptoms, CAT_activity, and CAT_impact. In the factorial analysis, a correlation matrix was used to correlate the three dimensions created in the CAT with the three dimensions of the SGRQ, classifying the CAT questions that had a (bilateral) statistical < 0.01 with some of the SGRQ spheres. In order to test the construct created, the Pearson correlation coefficient was measured between the means of the newly created spheres (CAT_sintomas, CAT_activity, CAT_impact) with existing ones (SGRQ_symptoms, SGRQ_activity, SGRQ_impact). The correlation coefficients were interpreted as follows: r < 0.10 (no correlation); r = 0.10 − 0.29 (weak correlation); r = 0.30 − 0.49 (moderate correlation); and r ≥ 0.50 (strong correlation)^(^ ^36^ ^)^. To estimate the reliability of each of the questionnaires of the sample studied, the internal consistency was analyzed through the Cronbach's Alpha. The agreement between the two questionnaires was measured through the Bland Altman method, which measures the degree of agreement between the final result of two questionnaires to see whether they behave similarly in the same individuals. To perform the calculation, it was considered necessary to multiply the CAT score by 0.25 to make it directly comparable with the total SGRQ score^(^ ^27^ ^)^. In order to observe the differences between the health status of the patient and the total scores of the questionnaires, associations were established between the control variables and the general score of the questionnaire. Tthe Student's t test was applied in the case of comparisons of two groups and Anova in the case of comparisons of three or more groups. All p values were reported for interpretation, considering statistically significant p values < 0.05. The statistical analyses were performed in the SPSS v.23 and the Epitat v4.2 for Windows.

The study was approved by the Bioethics and Research Committee of the HGUCS and by the Deontological Commission of *Universitat Jaume I.* It was carried out following the rules specified in the Declaration of Helsinki. The treatment of the data was adjusted to the provisions of the Spanish Organic Law on Protection of Personal Data, 15/1999, of December 13, and of Law 41/2002, of November 14, on basic regulations about patient autonomy and rights and information regarding clinical information and documentation. All the study participants signed a consent form to participate in the study.

## Results

A total of 466 patients were admitted for exacerbation of COPD in the HUGC, where 310 were excluded due to physical or mental disabilities (n = 66), terminal condition (n = 85), previous inclusion (n = 66), existence of language barrier (n = 9), or patients who had the criteria but rejected the invitation to share in the study (n = 84). Finally, a total of 156 patients met the inclusion criteria, but 153 (98.1%) were selected because of the presence of items left incomplete in the questionnaire. The majority were male, 79.1% (n = 121), with a mean age of 73.7 ± 9.8 SD years and primary education 48.4% (n = 74). In the clinical profile, 43.8% (n = 63) presented grade III level of dyspnea. The more prevalent signs and symptoms were sputum, 75.2% (n = 115), followed by cough, 60.8% (n = 93), and the need to sleep in the sitting position, 58.2% (n = 89). Of the mental health pathologies, the most prevalent was depression, 24.2% (n = 37) of the probable cases. Regarding the basic activities of daily living, 49.0% (n = 75) were severely dependent and 20.9% (n = 32) were moderately dependent. A summary of the characteristics of the sample is presented in Table 1.

**Table 1:** Characteristics of the sample according to the control variables. Castellón de la Plana, Comunidad Valenciana, Spain, 2014, 2015, 2016

Sociodemographic		
Age		73.7 ± (DP*9.8)
Sex		
	Male	121 (79.1%)
	Female	32 (20.9%)
Schooling		
	None	26 (17.0%)
	Primary school	74 (48.4%)
	Secondary school	42 (27.5%)
	College	11 (7.2%)
**Clinical variables**		
Dyspnea (MRC†)		
	0	2 (1.3%)
	I	6 (3.9%)
	II	30 (19.6%)
	III	67 (43.8%)
	IV	43 (28.1%)
Cough		93 (60.8%)
Sputum		115 (75.2%)
Wheezing		73 (47.7%)
Daytime drowsiness		47 (30.7%)
Fever		24 (15.7%)
Edemas		57 (37.3%)
Need to sleep in sitting position		89 (58.2%)
Pain (EVA‡)		1.4 ± (2.3)
**Psychological variables**		
Anxiety		
	No cases	39 (25.5%)
	Doubtful case	85 (55.6%)
	Probable case	29 (19.0%)
Depression	
	No cases	37 (24.2%)
	Doubtful case	79 (51.6%)
	Probable case	37 (24.2%)
**Daily life activities**		
Barthel Index		
	Total dependence	9 (5.9%)
	Severe dependence	75 (49.0%)
	Moderate dependence	32 (20.9%)
	Mild dependence	8 (5.2%)
	Independence	29 (19.0%)

* SD = Standard deviation;

† MRC = Medical Research Council;

‡ EVA = Visual Analog Scale

A factorial analysis was performed to classify the eight questions of the CAT according to the SGRQ spheres, which resulted in three spheres in the CAT. Thus, the sphere of symptoms of CAT presented a greater factorial load in the following items: cough, phlegm and oppression; the sphere of CAT_sintomas refered to a greater factorial load on the following items: climbing stairs and performing household activities; and finally the sphere CAT_impact gathered a greater factorial load on the items: confidence leaving home, no problem to sleep, and energy. Table 2 shows the correlation matrix of the spheres of the two questionnaires.

**Table 2:** Matrix of correlations of the questions of the Chronic Obstructive Pulmonary Diseases Assessment Test with the spheres of the St. George Respiratory Questionnaire. Castellón de la Plana, Comunidad Valenciana, Spain, 2014, 2015, 2016

	SGRQ* Symptoms	SGRQ* Activity	SGRQ* Impact
CAT† cough	0.316‡	0.095	0.201
CAT† phlegm	0.436‡	0.187	0.253
CAT† oppression	0.511‡	0.342	0.380
CAT† climbing stairs	0.458	0.537‡	0.509
CAT† household activities	0.466	0.486‡	0.491
CAT† confidence leaving home	0.474	0.461	0.565‡
CAT† sleep	0.528	0.455	0.539‡
CAT† energy	0.504	0.545	0.562‡

* SGRQ = St. George Respiratory Questionnaire;

† CAT = Chronic Obstructive Pulmonary Diseases Assessment Test;

‡ Spheres showing correlation p < 0.05

The Pearson correlation coefficient showed a correlation between the new spheres created in the CAT and the existing ones of the SGRQ. A strong correlation was obtained globally and in the spheres of activity and impact, as well as a moderate correlation in the sphere of symptoms, as shown in Table 3. It was also observed that the two questionnaires presented adequate internal consistency in the sample studied, with Cronbach's alpha coefficients of 0.843 for the SGRQ and 0.799 for the CAT (Table 3).

**Table 3:** Relationship between the spheres created from the Chronic Obstructive Pulmonary Disease Assessment Test with the spheres of the St George Respiratory Questionnaire. Castellón de la Plana, Comunidad Valenciana, Spain, 2014, 2015, 2016

	Correlation
CAT* symptoms	0.444‡
SGRQ* symptoms	
CAT* activity	0.591‡
SGRQ* activity	
CAT* impact	0.637‡
SGRQ* impact	
Total CAT*	0.628‡
SGRQ† total	
	**Cronbach's Alpha**
Total CAT*	0.799
SGRQ† total	0.843

* CAT = Chronic Obstructive Pulmonary Diseases Assessment Test;

† SGRQ = St George Respiratory Questionnaire;

‡ Spheres showing correlation p < 0.01

**Figure 1:**
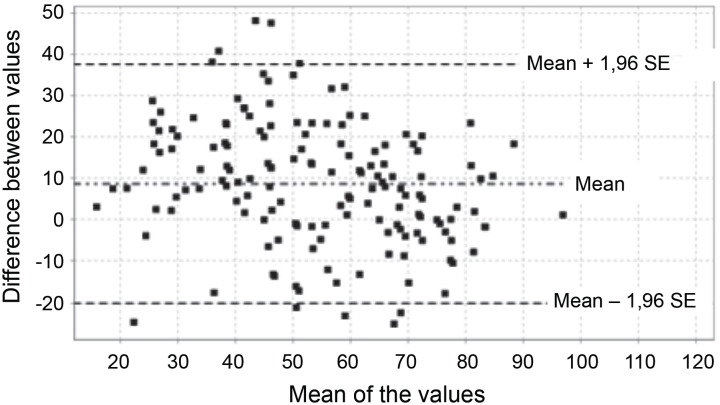
Bland and Altman total scores of the St. George Respiratoy Questionnaire and Chronic Obstructive Pulmonary Diseases Assessment Test. Castellón de la Plana, Comunidad Valenciana, Spain, 2014, 2015, 2016

The Bland and Altman graph (Figure 1) showed that the mean scores of each of the questionnaires were within the limits of agreement, confirming the agreement between the two questionnaires.

When correlating the global scores of the two questionnaires with the clinical variables, all variables were statistically significant in the two questionnaires, with the exception of cough and sputum, which showed statistical significance only in the CAT (p < 0.01), but not in the SGRQ (p = 0.129) and (p = 0.221). Table 4 shows the results obtained from the correlation between the two questionnaires.

**Table 4:** Bivariate analysis of the p value obtained between the final score of the questionnaire and the control variables. Castellón de la Plana, Comunidad Valenciana, Spain, 2014, 2015, 2016

	CAT*	SGRQ†
Dyspnea	0.000‡	0.000‡
Pain	0.004‡	0.001
Anxiety	0.000‡	0.000‡
Depression	0.000‡	0.000‡
Barthel Index	0.000‡	0.000‡
Cough	0.000‡	0.129
Sputum	0.002‡	0.221
Wheezing	0.000‡	0.000‡
Daytime drowsiness	0.001‡	0.000‡
Fever	0.547	0.232
Edemas	0.047‡	0.001‡
Need to sleep in sitting position	0.017‡	0.003‡

* CAT = Chronic Obstructive Pulmonary Diseases Assessment Test;

† SGRQ = St George Respiratory Questionnaire;

‡ Items that presented statistical significance.

## Discussion

The profile of the study sample was predominantly male, aged 73 years, and with primary schoold. These characteristics are similar to epidemiological studies carried out with COPD patients in the Spanish territory (IBEREPOC)^(^ ^37^ ^–^ ^38^ ^)^ or international studies^(^ ^39^ ^–^ ^40^ ^)^. Thus, patients presented a high number of signs and symptoms, such as dyspnea, sputum or cough, which were related to respiratory infections of exacerbations in COPD^(^ ^41^ ^)^, and the reason for the invitation to share in the study.

The CAT questionnaire, as well as the SGRQ, presented an internal reliability above 0.7, similar to another review^(^ ^25^ ^)^ on the attributes of the two questionnaires. Thus, the existence of correlation between the results of the two questionnaires has also been valued by several researchers^(^ ^13^ ^)^ and even a correlation of the SGRQ spheres with the total CAT result has been reported^(^ ^26^ ^)^, but no study correlated all questions with the SGRQ spheres. There was a correlation in the questions attributed to the sphere of symptoms, activity or impact of the SGRQ with the questions related to these items in the CAT.

Similarly, the results of this study demonstrated that HRQOL in COPD patients is associated with dyspnea^(^ ^7^ ^,^ ^42^ ^–^ ^46^ ^),^ pain^(^ ^7^ ^),^ anxiety and depression^(^ ^7^ ^,^ ^45^ ^,^ ^47^ ^)^, limitation in the performance of activities^(^ ^48^ ^)^, wheezing, daytime drowsiness, edema, and the need to sleep in the sitting position. It is important to highlight the increased sensitivity of the CAT in comparison to the SGRQ to detect cough and sputum, while maintaining the same sensitivity to detect the remaining variables. Thus, the use of the CAT questionnaire is considered better in patients with COPD exacerbation in the hospital setting, particularly in view of the easier and less time-consuming completion^(^ ^26^ ^)^. However, both questionnaires were considered sensitive when evaluating HRQOL in patients with COPD exacerbation in the hospital environment and in patients with stable COPD in the primary care setting, as identified in other studies^(^ ^22^ ^)^.

Finally, another aspect that favors the application of the CAT over the SGRQ is the filling time. The SGRG is more extensive and presents complex scoring algorithms, making its use in clinical practice to become ordinary and the repeated evaluation to become inadequate; in many cases, it is necessary to help patients complete it correctly^(^ ^49^ ^)^. The mean time to complete the CAT is 107 seconds, compared to 578 seconds of completion required by the SGRQ^(^ ^23^ ^)^. In hospital environments, the use of short questionnaires that facilitate information and improve communication between patients and health personnel is considered necessary^(^ ^50^ ^–^ ^51^ ^)^.

The main limitation of the present study is the nature of the data, because the study was not designed to verify the effectiveness of the SGRQ and the CAT on quality of life in hospitalized patients with severe COPD exacerbation. Therefore, data such as retests are missing despite the fact that if patients have data in three months, this information was rejected because patients do not present the same conditions, acting in the education program as a confounding factor. Another aspect that would be interesting to evaluate is the filling time of each of the questionnaires whose data were not studied.

## Conclusions

The CAT scores were correlated with the ones of the SGRQ, in total, according to spheres and questions. Both questionnaires have high internal consistency in patients admitted for exacerbation of COPD in the hospital setting, and the CAT is more sensitive in detecting changes in the HRQOL if the patient has cough and sputum only.

Therefore, the CAT is a reliable and accurate tool to be used in COPD patients with exacerbation of the problem in hospital settings, requiring a shorter time than the SGRQ.

The evaluation of the HRQOL in COPD patients is a good indicator of severity, and the onset of a new exacerbation and mortality. Their routine assessment is necessary for better disease monitoring in order to assess the impact of the disease and the effectiveness of the treatment for the performance of activities of daily living. The use of CAT will facilitate routine evaluation for physicians, nurses, physical therapists, and other health professionals in the hospital setting. Admission is the time of greatest need for follow-up necessary to control the efficacy of the administered treatments, with nursing being one of the groups which most uses the two questionnaires.
